# Phospholipases D1 and D2 Suppress Appetite and Protect against Overweight

**DOI:** 10.1371/journal.pone.0157607

**Published:** 2016-06-14

**Authors:** Jonathan Trujillo Viera, Rabih El-Merahbi, Bernhard Nieswandt, David Stegner, Grzegorz Sumara

**Affiliations:** 1 Rudolf Virchow Center for Experimental Biomedicine University of Würzburg, Josef-Schneider-Str. 2, Haus D15, D-97080 Würzburg, Germany; 2 Institute of Experimental Biomedicine, University Hospital of Würzburg, Josef-Schneider-Str. 2, Haus D15, D-97080 Würzburg, Germany; Institut d'Investigacions Biomèdiques August Pi i Sunyer, SPAIN

## Abstract

Obesity is a major risk factor predisposing to the development of peripheral insulin resistance and type 2 diabetes (T2D). Elevated food intake and/or decreased energy expenditure promotes body weight gain and acquisition of adipose tissue. Number of studies implicated phospholipase D (PLD) enzymes and their product, phosphatidic acid (PA), in regulation of signaling cascades controlling energy intake, energy dissipation and metabolic homeostasis. However, the impact of PLD enzymes on regulation of metabolism has not been directly determined so far. In this study we utilized mice deficient for two major PLD isoforms, PLD1 and PLD2, to assess the impact of these enzymes on regulation of metabolic homeostasis. We showed that mice lacking PLD1 or PLD2 consume more food than corresponding control animals. Moreover, mice deficient for PLD2, but not PLD1, present reduced energy expenditure. In addition, deletion of either of the PLD enzymes resulted in development of elevated body weight and increased adipose tissue content in aged animals. Consistent with the fact that elevated content of adipose tissue predisposes to the development of hyperlipidemia and insulin resistance, characteristic for the pre-diabetic state, we observed that *Pld1*^*-/-*^ and *Pld2*^*-/-*^ mice present elevated free fatty acids (FFA) levels and are insulin as well as glucose intolerant. In conclusion, our data suggest that deficiency of PLD1 or PLD2 activity promotes development of overweight and diabetes.

## Introduction

Excessive caloric intake and/or insufficient nutrients utilization result in increased fat accumulation in adipose tissue, adipocytes hypertrophy and hyperplasia and are often associated with induction of low grade inflammation of adipose tissue. This leads to increased levels of lipids and inflammatory mediators and promotes peripheral insulin resistance which ultimately leads to T2D [1]. Caloric intake and energy consumption are tightly regulated by hormones and nervous system. Signals evoked by hormones, nutrients and afferent neurons are coordinated in the hypothalamus to control energy balance.

Phospholipase D enzymes promote hydrolysis of the phosphodiester bond of phosphatidylcholine, generating phosphatidic acid and choline. PA is the intracellular lipid mediator of PLDs’ biological functions. However, PA is tightly regulated and can be converted to other potentially bioactive lipids, including diacylglycerol, lysophosphatidic acid and cyclic phosphatidic acid (CPA) [2, 3]. PLDs have been described in multiple organisms including mammals. Based on homology, six members of PLD family (PLD1 –PLD6) have been identified. However, only PLD1, PLD2 and PLD6 possess enzymatic activity [4]. PLD1 and PLD2 are associated with intracellular membranes and are expressed in nearly all cell types in the body [4].

Activity of PLDs is regulated by phosphatidylinositol-4,5-bisphosphate, protein kinase C, ADP Ribosylation Factor and Rho family GTPases [2, 3]. By generating PA, PLD1 and PLD2 regulate multiple signaling cascades. PA directly associates to mammalian target of rapamycin (mTOR) and promotes its activation [5]. PLDs also directly regulate transcription by inhibiting the action of peroxisome proliferator-activated receptor γ (PPARγ) [6]. Moreover, PA directly promotes vesicle fission and fusion. Therefore, PLD1 and 2 are crucial enzymes regulating vesicle trafficking, secretion, endocytosis as well as receptor signaling [7, 8]. PLDs and PA seem to be also implicated in the survival, growing and invasion of tumors [9, 10] probably by activating mTOR [5], stimulating cell migration [9, 11], promoting autophagy [12] and tumor vascularization [9, 10]. In addition, PLDs might promote skeletal muscle regeneration [13] and take part in some neurodegenerative disorders [14]. PLD activity increases under stimulation of platelets [15] and is implicated in dense granules exocytosis [16].

Mice deficient for PLD1, PLD2 or both enzymes are viable, fertile and do not show any obvious phenotype. However, deletion of PLD1 and PLD2 in mice is protective against arterial thrombosis and ischemic stroke [17]. Moreover, recent studies revealed that absence of these isoforms might impair cognitive functions in mice [18]. However, deletion of PLD2 in a mouse model for development of Alzheimer disease ameliorates symptoms of this neurodegenerative disorder [19].

Despite the fact that both, PLD1 and PLD2, enzymes were shown to contribute to regulation of the major signaling cascades implicated in regulation of metabolic homeostasis (mTOR and PPARγ), their impact on these processes has not been evaluated so far.

Here, we utilized mice deficient for PLD1 and PLD2 to assess the impact of these enzymes on regulation of metabolic homeostasis. We showed that mice lacking PLD2 and, to a lesser extent, PLD1 develop overweight when aged. This phenotype was caused by an increased food intake in both mice lines. Additionally, *Pld2*^*-/-*^ mice presented reduced energy expenditure. Consistent with the notion that obesity promotes development of T2D, we found that mice deficient for PLD1 or PLD2 are insulin resistant and glucose intolerant.

## Materials & Methods

### Mice

*Pld1*^*-/-*^ and *Pld2*^*-/-*^ mice used in this study have been previously described [17, 20]. Mice were housed in cages (max. 5 mice per cage) containing sawdust bedding and a nest made from paper tissues. The cages were kept at 23°C and underwent a 12-hour light phase followed by a 12-hour dark phase. Mice were fed a chow-control diet ad libitum and had unlimited access to water throughout the study. Mice were maintained following the guidelines for animal care and welfare according to Tierschutz-Versuchstierverordnung with the approval of the district government of Lower Franconia (Bezirksregierung Unterfranken.). Euthanasia was performed by cervical dislocation, in a separate area away from other animals and all efforts were made to minimize suffering. No animals died or became ill during the development of this research. All the animal studies were approved by the animal welfare committee of district government of Lower Franconia (Bezirksregierung Unterfranken. Az: 55.2–2531.01-124/13), in accordance with guidelines of the European Union.

### Weight and body composition

Measurement of body weight was done weekly from birth to week 20. Body composition, including fat mass, lean mass and free fluids content, was analyzed by Nuclear Magnetic Resonance (NMR) using a Bruker’s Minispec LF50 body composition analyzer according to the manufacturer’s instructions (Bruker Optik, Ettlingen, Germany). For dissection of organs, mice were sacrificed by cervical dislocation. Three adipose depots (EpiWAT, SubWAT and BAT), quadriceps, spleen, liver, hypothalamus, pancreas, heart and kidneys were dissected, weighted and quickly frozen in liquid nitrogen.

### Insulin and glucose tolerance test

An insulin tolerance test (ITT) was done 16 weeks after birth. Mice were fasted 4 hours prior to experiment. For this test, glucose measurements were done before and after intraperitoneal injection of insulin solution human (19278, Sigma-Aldrich, Germany) in Dulbecco´s phosphate buffered saline (14190–094, Life technologies Invitrogen). 0.5 units/kg of insulin were injected according to body weight. A blood drop was collected from the tail tip directly onto the test strip for the blood glucose measurement. Accu-chek Inform II strips (Roche, Germany) were used in a glucometer Accu-chek Performa (Roche, USA).

A glucose tolerance test (GTT) was done at 18 weeks after birth. Mice were fasted 13 hours prior to experiment. For this test, glucose measurements were done before and after intraperitoneal injection of 20% sterile glucose solution (HN06.2, Carl Roth, Germany) in Dulbecco´s phosphate buffered saline (14190–094, Life technologies, Invitrogen). 2 g/kg of glucose was injected according to body weight. The blood collection and glucose measurement were performed as for ITT. A restraining device was used in order to collect the blood samples for GTT and ITT.

### Analysis of plasma lipid and hormones

Blood samples were collected by tail incision using a restraining device. FFA plasma content was analyzed using a Wako NEFA-C kit (Wako NEFA-HR2) as described in manufacturer manual. Glycerol and triglycerides in plasma were analyzed using free glycerol reagent (F6428, Sigma, Germany). Insulin plasma content was measured using suspension bead array immunoassay kits following manufacturer's specifications (Bio-Plex Pro Mouse Diabetes Assay, Biorad, USA) on a Bio-Plex series 100 instrument (Biorad, USA).

### Energy metabolism measurement

For food intake, oxygen consumption and carbon dioxide dissipation measurements, mice were single housed and monitored by using a laboratory animal monitoring system PhenoMaster (TSE Systems) as it was previously described [21]. All mice were maintained on a 12 h light–dark cycle at 22°C with unrestricted access to food and water. Energy expenditure and respiratory exchange rate were calculated from indirect calorimetry data as described [22]. Mice were familiarized to single housing and drinking bottles for at least 48 h before metabolic phenotyping. Then, measurements were performed in a 10 minutes interval during five consecutive days. Data of the first 24 h were excluded from all analyses thus the results include data over four light/dark cycles. The results shown in the scatter plots are an average of each hour and the bar charts show the average of each light/dark cycle.

### Refeeding after fasting

Mice were single housed and monitored by using a laboratory animal monitoring system PhenoMaster (TSE Systems) as it was previously described [21]. All mice were maintained on a 12 h light–dark cycle at 22°C. After 6 days, mice were fasted overnight for 16 hours with free access to water. Food was restored after the fasting period and measurements were performed each 10 minutes for 24 hours.

### Quantitative real time PCR (qPCR)

RNA was isolated using QIAzol reagent (QIAzol lysis reagent, 79306, QIAGEN, USA) according to the manufacturer’s instructions. Total RNA was transcribed into complementary DNA using First Strand cDNA Synthesis Kit (K1612, Thermoscientific, Lithuania) according to the manufacturer’s instructions. qPCR was performed using the FastStart Universal SYBR Green Master (Roche) and the primers included in S1 Table. Primer list for the qPCR analysis.

### Statistical analysis

For multiple comparison one way analyzes of variance (ANOVA) followed by the Post hoc Tukey test was used. Significance was accepted at the level of 0.05.

## Results

### *Pld1*^*-/-*^ and *Pld2*^*-/-*^ mice present increased body weight and body fat content

**Fig 1:**
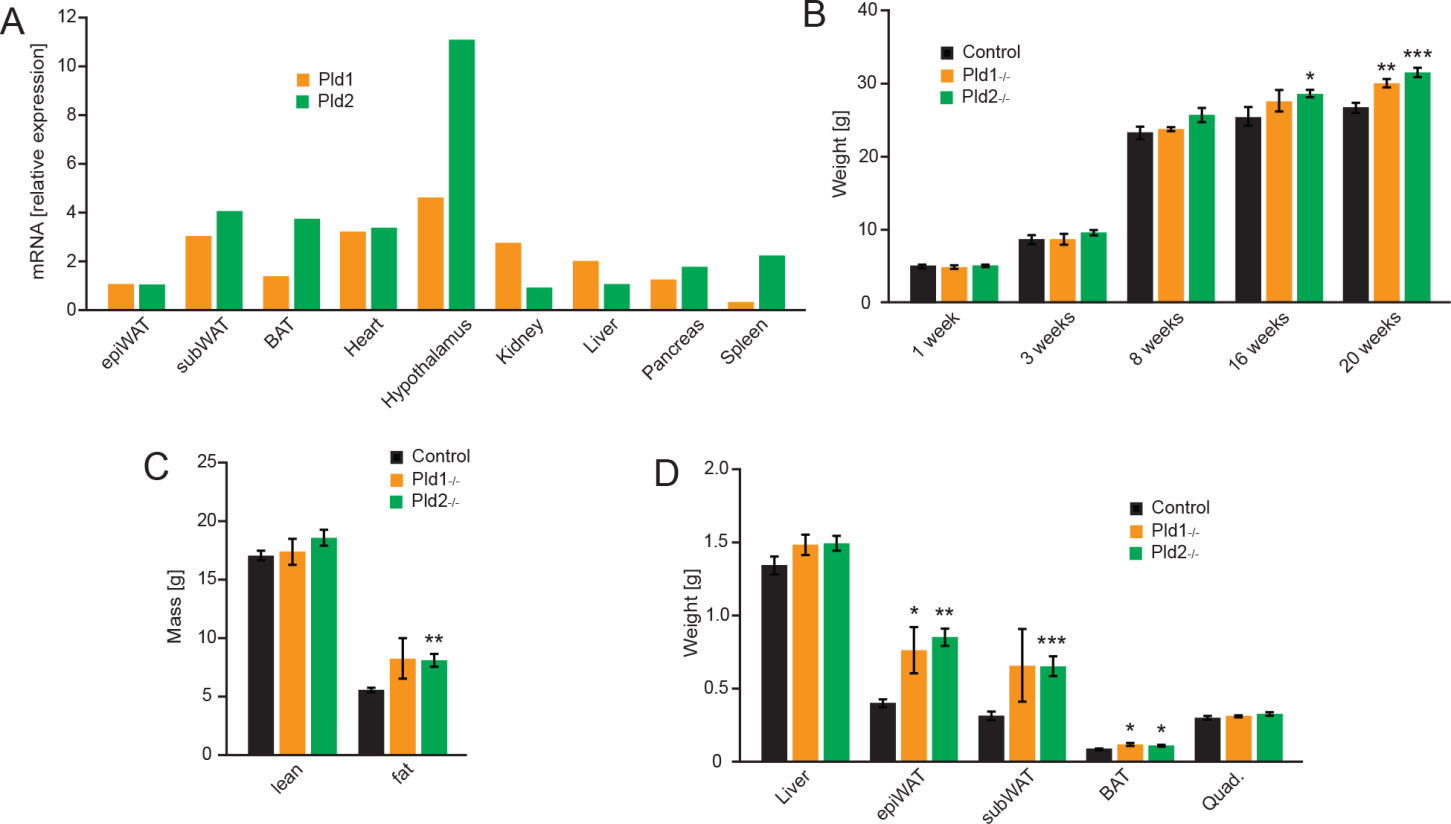
*Pld1 and Pld2* deletion promotes accumulation of body mass and fat. A) Relative expression of *Pld1* and *Pld2* mRNA in different organs of C57BL/6J mice. B) Body weight of mice at 1, 3, 8, 16 and 20 weeks after birth. C) NMR analysis of lean and fat mass of 20 weeks old mice. D) Weight of indicated organs dissected from 20 weeks old mice. Data represented as mean +/- S.E.M., n = 12 males for control (black), n = 7 males for *Pld1*^*-/-*^ (orange), n = 10 males for *Pld2*^*-/-*^ (green). *p<0.05, **p<0.01, ***p<0.001

Both isoforms of phospholipase D have been reported to be ubiquitously expressed in different organs and tissues [4, 23]. In order to corroborate these findings, we analyzed mRNA expression of both PLD isoforms in organs of wild type mice by qPCR. Consistent with previous observations, expression of *Pld1* and *Pld2* was found in all the organs tested (white and brown adipose tissue, heart, hypothalamus, kidney, liver pancreas and spleen) (Fig 1A). Of note, we observed a remarkably high hypothalamic expression of *Pld2* (Fig 1A).

PLD1 and PLD2 generate phosphatidic acid that is crucial for selected signaling pathways [4]. PA regulates several cellular signaling cascades like mTOR and PPARγ which are implicated in whole-body metabolic homeostasis. PA stimulates mTOR activity in the cells [2, 4] which promotes growth, protein and lipid synthesis and suppress autophagy [24]. In addition, a derivative of PA, cyclic phosphatidic acid, suppresses a central transcription factor promoting lipid synthesis and adipogenesis, PPARγ [6, 25]. Moreover, both PLD isoforms are expressed in major organs implicated in regulation of metabolic homeostasis. However, the impact of PLD1 and PLD2 on regulation of metabolism has not been elucidated so far. To investigate the function of these enzymes, we utilized mice deficient for PLD1 (*Pld1*^*-/-*^) or PLD2 (*Pld2*^*-/-*^). Consistent with the previous report, we observed that up to 3 weeks of age, weight of *Pld1*^*-/-*^ and *Pld2*^*-/-*^ mice was indistinguishable from control animals (Fig 1B). However, at 20 weeks of age, *Pld1*^*-/-*^ and *Pld2*^*-/-*^ mice accumulated significantly more body mass than corresponding controls (Fig 1B). Next, to determine body composition of *Pld1*^*-/-*^ and *Pld2*^*-/-*^ mice, we utilized nuclear magnetic resonance (NMR). At 20 weeks of age mice deficient for PLD2, presented markedly and significantly increased fat content in the body compare to control animals (Fig 1C), while *Pld1*^*-/-*^ mice showed clear tendency towards increased body fat content which, however, did not reach significance. Of note, lean mass content was not altered by deletion of *Pld1* or *Pld2* (Fig 1C). Consistent with these observations, dissection of 20 weeks old *Pld1*^*-/-*^ and *Pld2*^*-/-*^ mice reveled increased weight of different body fat depots but no difference in the weight of muscles or liver compared to the control animals (Fig 1D).

Taken together, these findings suggest that PLD1 and PLD2 enzymes suppress age-dependent body weight and adipose tissue acquisition.

### PLD1 and PLD2 suppress food intake

**Fig 2:**
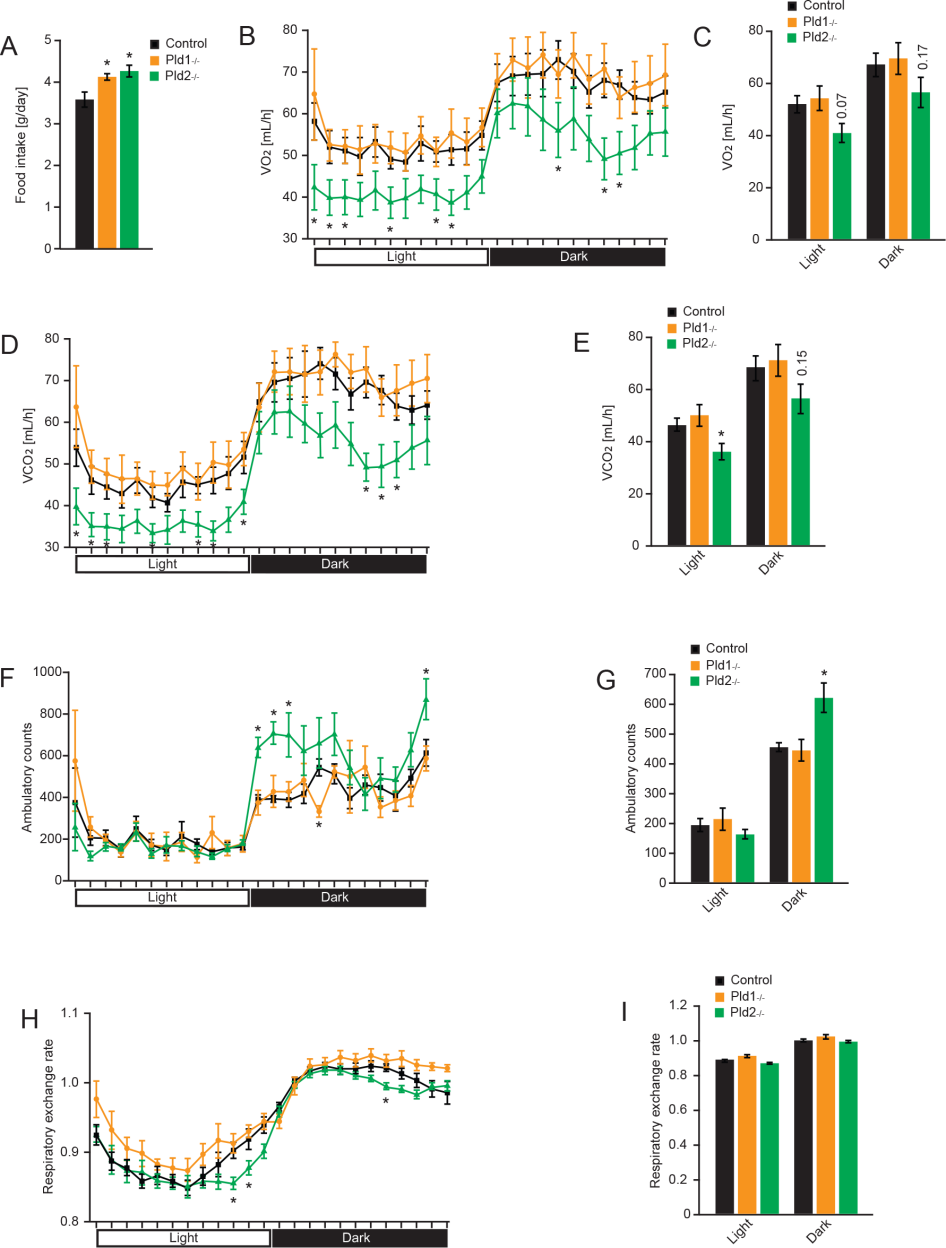
Deletion of *Pld1* or *Pld2* promotes food intake. A) 24 hours cumulative food intake of 20-weeks old mice with indicated genotypes. B) Oxygen consumption of mice with indicated genotypes at different time of the day. C) Average oxygen consumption during light and dark phase. D) Carbon dioxide production of mice with indicated genotypes at different time of the day. E) Average carbon dioxide during light and dark phase. F) Voluntary movements of mice with indicated genotypes at different time of the day. G) Average voluntary movements of mice during light and dark phase. H) Respiratory exchange rate of mice with indicated genotypes at different time of the day. I) Average respiratory exchange rate of mice during light and dark phase. Data represented as mean +/- S.E.M., n = 6 males for control (black), n = 4 males for *Pld1*^*-/-*^ (orange), n = 6 males for *Pld2*^*-/-*^ (green). *p<0.05, **p<0.01, ***p<0.001

Abnormal body weight and fat acquisition is usually caused by imbalance between food intake and energy expenditure. To investigate the impact of PLDs deletion on appetite, energy expenditure and physical activity of the mice, we utilized PhenoMaster home cage system. Deletion of *Pld1* or *Pld2* resulted in increased cumulative food intake compare to control animals (Fig 2A). Whole body oxygen consumption rates (Fig 2B and 2C) and carbon dioxide dissipation (Fig 2D and 2E) were significantly decreased in *Pld2*^*-/-*^ mice over several time points of the light and dark phases, reflecting lower energy expenditure. However, despite decreased energy expenditure in *Pld2*^-/-^ mice, these mice recorded an increase in their activity over the night phase compared to the controls (Fig 2F and 2G). On the contrary, mice deficient for PLD1 did not present alteration in oxygen consumption, carbon dioxide dissipation and voluntary movements (Fig 2B–2G). The respiratory exchange ratio was then calculated to determine the main source of fuel being metabolized by the mice. Data show no alteration of free fatty acids/carbohydrates utilization in *Pld1*^*-/-*^ mice compared to the controls (Fig 2H and 2I). However, deletion of *Pld2* might increase consumption of fat over carbohydrates at the specific times of the day as the respiratory exchange ratio was slightly but significantly decreased at three time points of the 24 h cycle of measurements. In summary, deletion of PLD enzymes promotes positive energy balance in mice which explains increased age-dependent accumulation of fat in these animals.

### *Pld1*^*-/-*^ and *Pld2*^*-/-*^ mice present normal satiety response

**Fig 3:**
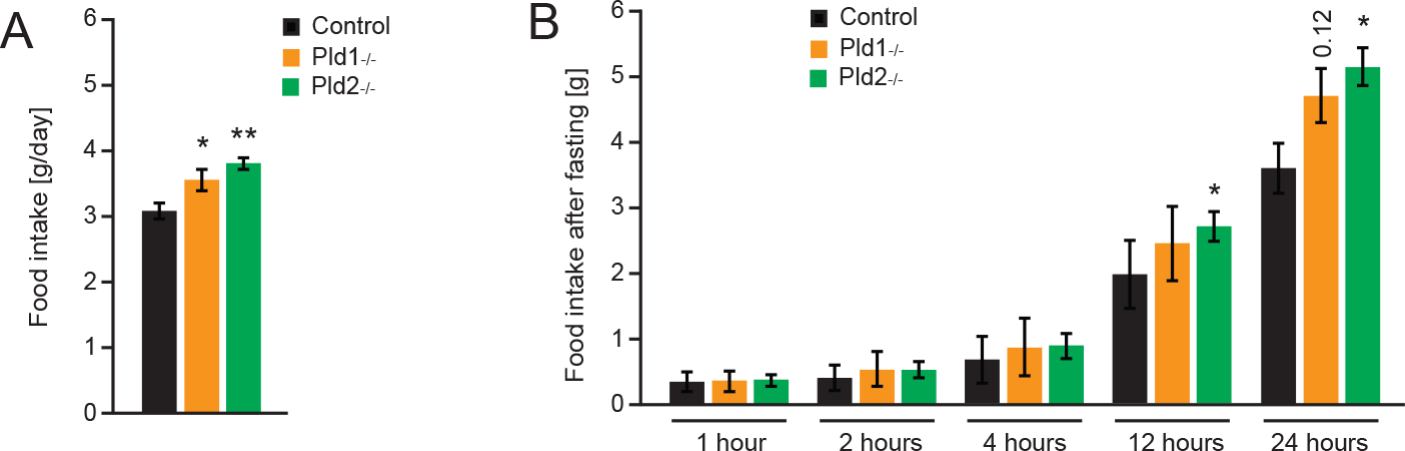
Deletion of *Pld1* or *Pld2* does not affect satiety response. A) 24 hours cumulative food intake of 20-weeks old mice with indicated genotypes. B) Food intake at different time-points after overnight fasting of 20-weeks old mice with indicated genotypes. Data represented as mean +/- S.E.M., n = 6 females for control (black), n = 4 females for *Pld1*^*-/-*^ (orange), n = 6 females for *Pld2*^*-/-*^ (green). *p<0.05, **p<0.01, ***p<0.001

In order to test whether the hyperphagia was due to a long or a short-term stimulus, a refeeding after fasting experiment was performed. Food intake was monitored before the fasting period and Fig 3A shows for females the same tendency in the feeding behavior as previously showed for males in Fig 2A. After the fasting period the food intake was measured as shown in Fig 3B. *Pld1*^***-/-***^ and *Pld2*^*-/-*^ mice exhibit a normal response to starvation during the first hours after refeeding but maintain the long-term hyperphagia after the 24 hours period. These results show that the increased food intake of the PLD1 or PLD2 depleted mice is not due to a defect in satiety mechanism but it is more likely to be due to adiposity signals.

### Deletion of *Pld1* or *Pld2* results in an altered expression of genes involved in regulation of food intake in hypothalamus

**Fig 4:**
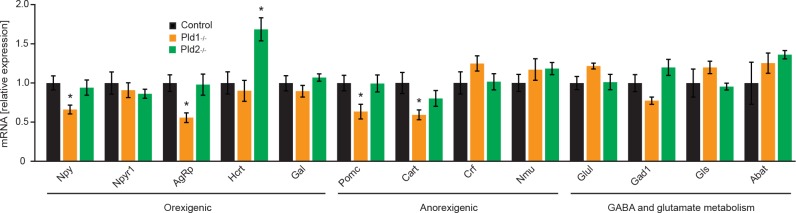
mRNA expression in hypothalamus of neuropeptides controlling food intake. Relative expression of mRNA in hypothalamus of *Pld1*^*-/-*^, *Pld2*^*-/-*^ and control mice. The analyzed targets are presented as those with known orexigenic effect: neuropeptide Y (Npy), neuropeptide Y receptor 1 (Npyr1), Agouti Related Neuropeptide (AgRp), Hypocretin (Hcrt), Galanin (Gal); those with anorexigenic effect: Pro-opiomelanocortin (Pomc), Cocaine-amphetamine-regulated transcript (Cart), Corticotropin-releasing factor (Crf), Neuromedin U (Nmu); and those involved in the metabolism of GABA and glutamate: Glutamate-ammonia ligase (Glul), Glutamate decarboxylase (Gad1), Glutaminase (Gls) and 4-aminobutyrate aminotransferase (Abat). Data represented as mean +/- S.E.M., n = 6 males for control (black), n = 5 males for *Pld1*^*-/-*^ (orange), n = 6 males for *Pld2*^*-/-*^ (green). *p<0.05, **p<0.01, ***p<0.001

Short- and long-term signals reflecting the metabolic state of the organism are integrated and processed in the hypothalamus. In there, orexigenic and anorexigenic neurons control the feeding behavior. Therefore, a qPCR analysis was performed to quantify the relative expression of different genes involved in regulation of food intake and in the metabolism of GABA and glutamate [26]. As shown in Fig 4, mice deficient for *Pld1* have a lower expression of the orexigenic neuropeptide Y (Npy) and agouti related neuropeptide (AgRp) but at the same time decreased expression of the anorexigenic pro-opiomelanocortin (Pomc) and cocaine-amphetamine-regulated transcript (Cart). On the other hand, *Pld2*^*-/-*^ mice present elevated levels of the well-known orexigenic Hypocretin (Hcrt) which can partially explain the increased food intake of this genotype. Different enzymes involved in GABA and glutamate metabolism were not altered at the mRNA level. This expression profile shows an alteration in different neuropeptides controlling food intake in hypothalamus of *Pld1*^*-/-*^ and *Pld2*^*-/-*^ mice.

### Free fatty acids in circulation are elevated in mice deficient for PLD1 or PLD2

**Fig 5:**
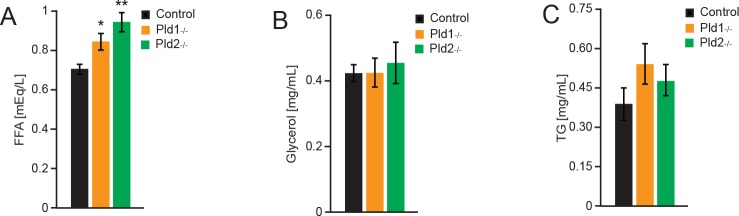
Free fatty acids in circulation are elevated in mice deficient for PLD1 or PLD2. A) Free fatty acids (FFAs), B) Glycerol, C) Triglycerides in circulation of 18-weeks old mice with indicated genotypes. Data represented as mean +/- S.E.M., n = 9 males for control (black), n = 6 males for *Pld1*^*-/-*^ (orange), n = 8 males for *Pld2*^*-/-*^ (green). *p<0.05, **p<0.01, ***p<0.001

High levels of free fatty acids are generally associated with increased adipose tissue and body weight. This elevation of FFA promotes peripheral insulin resistance [27]. To check if increased adiposity in PLD-knockout mice promotes elevation of lipids in circulation, blood samples were collected, analyzed and compared between the knockout mice and the controls. A significant elevation of FFA in circulation of *Pld1*^*-/-*^ and *Pld2*^*-/-*^ mice was observed (Fig 5A). Glycerol and triglycerides levels were not changed (Fig 5B and 5C).

### PLD1 or PLD2 deletion results in insulin resistance and glucose intolerance

**Fig 6:**
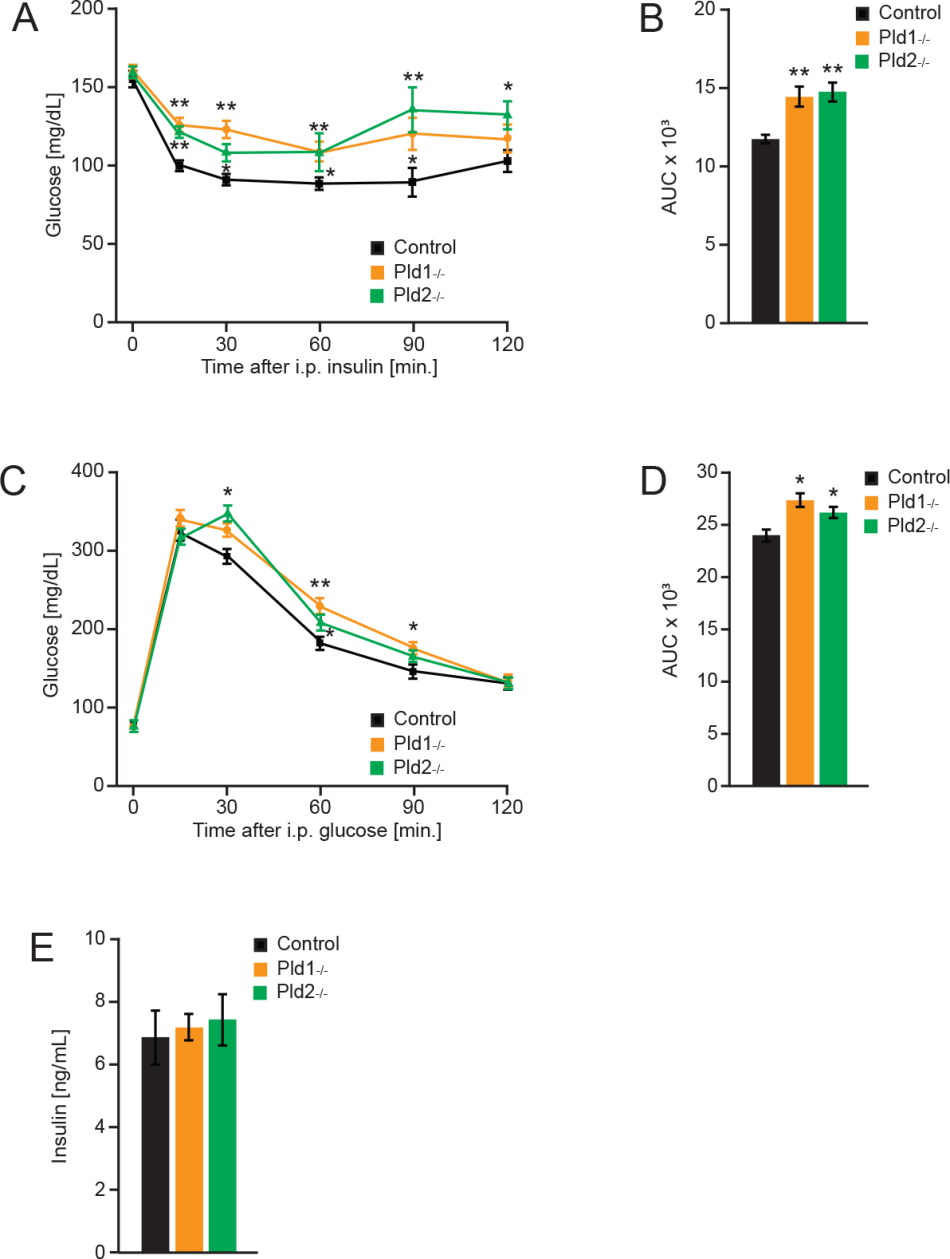
Deletion of Pld1 or Pld2 promotes insulin resistance and glucose intolerance. A) Insulin tolerance test in *Pld1*^*-/-*^, *Pld2*^*-/-*^ and control mice at 16 weeks old. n = 7 for control (black), n = 6 for *Pld1*^*-/-*^ (orange), n = 8 for *Pld2*^*-/-*^ (green). B) Area under the curve for the insulin tolerance test. C) Glucose tolerance test in *Pld1*^*-/-*^, *Pld2*^*-/-*^ and control mice at 18 weeks old. n = 12 for control (black), n = 10 for *Pld1*^*-/-*^ (orange), n = 12 for *Pld2*^*-/-*^ (green). D) Area under the curve for the glucose tolerance test. E) Insulin levels in circulation of mice with indicated genotypes at 20 weeks old. n = 8 males for control (black), n = 6 males for *Pld1*^*-/-*^ (orange), n = 8 males for *Pld2*^*-/-*^ (green). Data represented as mean +/- S.E.M., *p<0.05, **p<0.01, ***p<0.001

Insulin insensitivity is commonly developed as a consequence of increased body weight and high levels of FFA in circulation. It has been showed that high levels of FFA can impair insulin sensitivity in muscles and liver [27]. As shown in Fig 6A and 6B, mice deficient for PLD1 or PLD2 have a decreased insulin sensitivity. Both deletions leaded to higher glucose levels after insulin injection at different time points of the experiment, indicating impairment in insulin-stimulated glucose uptake. These results are supported by the increased adiposity and circulating free fatty acids showed before.

We further performed glucose tolerance test as it is an indicative measurement of glucose uptake, distribution and metabolism that correlates to the development of type 2 diabetes. After intraperitoneal administration of a specific dosage of glucose (2 g/kg of body weight), PLD1 or PLD2 deficiency resulted in an impairment of glucose uptake as it is demonstrated by elevated glucose concentrations in the bloodstream at different times of measurement (Fig 6D and 6E). These results are consistent with our previous observations showing the development of insulin intolerance when phospholipase D is absent. Of note, blood insulin levels were not changed in PLDs deficient mice (Fig 6E). Consequently, glucose intolerance in the absence of either of PLD isoforms is rather caused by insulin intolerance than insulin secretion.

Therefore, deficiency of PLD enzymes results in development of insulin resistance characteristic of pre-diabetic state.

## Discussion

In this study we showed that PLD1 and PLD2 suppress food intake. Furthermore, specifically PLD2, but not PLD1, promotes dissipation of energy. Consequently, mice lacking PLD1 or PLD2 develop increased body weight gain when aged. Moreover, increased adiposity and high lipid levels in circulation promote insulin resistance and glucose intolerance in both *Pld1*^*-/-*^ and *Pld2*^*-/-*^ animals.

Mice deficient for PLD1 and PLD2 consume 15 and 18% more food respectively (Fig 2A). Moreover, *Pld2*^*-/-*^ mice present decreased energy expenditure but increased voluntary movement during the dark phase (Fig 2B–2G). However, satiety mechanism is not affected in mice deficient for PLD1 or PLD2 (Fig 3B). We showed that PLD2 and, to a lesser extent, PLD1 are highly expressed in hypothalamus, a major center of coordination of satiety and hunger inputs. Analyzes of expression of neuropeptides implicated in regulation of food intake in hypothalamus, revealed that PLD1 and PLD2 regulate distinct set of genes in this organ. Mice deficient for PLD2 presents increased expression of Hcrt (also known as orexin) (Fig 4). Hcrt promotes food intake and voluntary movements of mice [28], therefore enhanced expression of this neuropeptide might partially explain phenotype observed in *Pld2*^*-/-*^ mice. On the other hand, Hcrt promotes energy expenditure [28]. Our results indicate that PLD2 deficiency results in reduced energy utilization. Therefore, PLD2 rather regulates energy expenditure by other mechanisms. Deletion of PLD1 results in reduction of anorexigenic neuropeptides Pomc and Cart. However, at the same time we observed reduction of orexigenic Npy and AgRp in *Pld1*^*-/-*^ mice (Fig 4). These results might indicate that, in *Pld1*^*-/-*^ mice, the decreased expression of Cart and Pomc might dominate on the effect of reduction in mRNA levels for Npy and AgRp.

PLD enzymes might modulate expression by regulating local concentration PA and its derivatives. In fact, cyclic phosphatidic acid, which is generated from PA in a PLD-dependent manner, suppresses PPARγ activity [6]. Interestingly, activation of PPARγ in central nervous system results in elevated food intake [29]. Therefore, increased transcriptional activity of PPARγ in the neurons of PLD1 and PLD2 deficient mice, might potentially explain increased food intake of these animals. Additionally, previous studies indicate that PA promotes mTOR activation [30]. The impact of mTOR activation on hypothalamic regulation of food intake has been a subject of intense investigation. Pharmacological inhibition of mTOR activity in anorectic POMC neurons of the hypothalamus results in enhanced food intake [31]. However, long term genetic activation of mTOR activity in POMC neurons (by deletion of tuberous sclerosis protein 1) also resulted in elevated food intake [32]. To our knowledge, impact of PPARγ or mTOR-dependent signaling on expression of neuropeptides in hypothalamus has not been investigated in detail. Therefore, we cannot correlate it with the expression patterns observed in *Pld1*^*-/-*^ and *Pld2*^*-/-*^ mice.

Also, based on our results we cannot conclude which of these signaling cascades is affected by the deletion of *Pld1* or *Pld2*. Since in our study we utilized mice deficient for the enzymes in the entire body we also cannot exclude the possibility that PLD-dependent inhibition of food intake originates outside of the central nervous system. However, our results indicate that elevated body weight and adiposity in mice deficient for PLD1 and PLD2 is caused by the increased food intake in these animals. In addition, elevation of PPARγ activity in the absence of PLD1 and PLD2 might also take place in the adipocytes, which also might contribute to the increase in body weight in these mice.

Elevated levels of FFA observed in mice lacking PLDs enzymes (Fig 5F) are most likely a result of the increased in adipose tissue content. Of note, elevation of FFA levels were correlated with peripheral insulin resistance and glucose intolerance in multiple studies (reviewed in [27]). Consistently, mice deficient for PLD1 and 2 are glucose and insulin intolerant as measured by insulin and glucose tolerance test (Fig 6A–6D). At this stage we cannot conclude which cell types/organs are resistant to insulin action in the absence of PLD1 or PLD2. To further investigate this issue and to ultimately establish the role of PLDs in regulation of insulin sensitivity, insulin action during hyperinsulinemic-euglycemic clamp needs to be assessed in *Pld1*^*-/-*^ and *Pld2*^*-/-*^ mice. Both PLD enzymes might be implicated in regulation of secretion as it has been previously reported [7, 8]. However, we showed that the levels of insulin, a major hormone regulating glucose homeostasis, are not affected by deletion of *Pld1* or *Pld2*.

Previous studies indicate that of deletion of *Pld1* and *Pld2* protects against development of arterial thrombosis and ischemic stroke [17, 20, 33]. Consequently, inhibitors of these enzymes have been proposed as a potential treatment for thrombotic diseases. PLDs inhibitors are also considered for treatment of cancer, multiple sclerosis, hypertension and several inflammatory disorders [4]. Our data indicates that the long-term usage of PLD inhibitors as a pharmacological treatment might cause potential side effects including increased body weight and peripheral insulin resistance.

## Supporting Information

S1 Data Raw data Fig 1A. (XLSX)

S2 Data Raw data Fig 1B. (XLSX)

S3 Data Raw data Fig 1C and 1D. (XLSX)

S4 Data Raw data Fig 2A. (XLSX)

S5 Data Raw data Fig 2B and 2C. (XLSX)

S6 Data Raw data Fig 2D and 2E. (XLSX)

S7 Data Raw data Fig 2F and 2G. (XLSX)

S8 Data Raw data Fig 2H and 2I. (XLSX)

S9 Data Raw data Fig 3A. (XLSX)

S10 Data Raw data Fig 3B. (XLSX)

S11 Data Raw data Fig 4. (XLSX)

S12 Data Raw data Fig 5. (XLSX)

S13 Data Raw data Fig 6A and 6B. (XLSX)

S14 Data Raw data Fig 6C and 6D. (XLSX)

S15 Data Raw data Fig 6E. (XLSX)

S1 Table Primer list for the qPCR analysis. (DOCX)
