# Supplementary material for: Phospholipases D1 and D2 Suppress Appetite and Protect against Overweight
Source: PLoS One. 2016 Jun 14;11(6):e0157607. doi: 10.1371/journal.pone.0157607 (PMC4907468; doi:10.1371/journal.pone.0157607)
Supplement: S1 Table — (DOCX) [file pone.0157607.s016.docx]

**S1 Table. Primer list for the qPCR analysis.**

| **Name** | **Symbol** | **Forward primer** | **Reverse primer** |
| --- | --- | --- | --- |
| Phospholipase D1 | **Pld1** | CATCGACAGCACCTCCAAC | GAGTTCTCCCACTCCGGTCT |
| Phospholipase D2 | **Pld2** | GTGCCACTGTGCAGGTCTTGAGG | GCAGAATAGCCTGGATGGAG |
| Neuropeptide Y | **Npy** | CTCCGCTCTGCGACACTAC | GGAAGGGTCTTCAAGCCTTGT |
| Neuropeptide Y receptor 1 | **Npyr1** | TGATCTCCACCTGCGTCAAC | ATGGCTATGGTCTCGTAGTCAT |
| Agouti Related Neuropeptide | **AgRp** | ATGCTGACTGCAATGTTGCTG | CAGACTTAGACCTGGGAACTCT |
| Hypocretin (orexin) | **Hcrt** | GTCGCCAGAAGACGTGTTC | GGTGGTAGTTACGGTCGGAC |
| Galanin | **Gal** | GGCAGCGTTATCCTGCTAGG | CTGTTCAGGGTCCAACCTCT |
| Pro-opiomelanocortin | **Pomc** | ATGCCGAGATTCTGCTACAGT | TCCAGCGAGAGGTCGAGTTT |
| Cocaine-amphetamine-regulated transcript | **Cart** | GCCAAGTCCCCATGTGTGAC | CACCCCTTCACAAGCACTTCA |
| Corticotropin-releasing factor | **Crf** | CCTCAGCCGGTTCTGATCC | GCGGAAAAAGTTAGCCGCAG |
| Neuromedin U | **Nmu** | GAGGGAGCTTTGCCGTATAGT | GATGCACAACAGAGGACACAA |
| Glutamate-ammonia ligase | **Glul** | TGAACAAAGGCATCAAGCAAATG | CAGTCCAGGGTACGGGTCTT |
| Glutamate decarboxylase | **Gad1** | CCGCCAGGTACTAAGCGAC | GCCCTCGGGCATTTTAATGAG |
| Glutaminase | **Gls** | GACAACGTCAGATGGTGTCAT | TGCTTGTGTCAACAAAACAATGT |
| 4-aminobutyrate aminotransferase | **Abat** | CTGAACACAATCCAGAATGCAGA | GGTTGTAACCTATGGGCACAG |
| Ribosomal protein L13A | **Rpl13a** | GCAGAATAGCCTGGATGGAG | GACCACCATCCGCTTTTTCTT |
